# Calprotectin and Platelet Aggregation in Patients with Stable Coronary Artery Disease

**DOI:** 10.1371/journal.pone.0125992

**Published:** 2015-05-13

**Authors:** Sanne Bøjet Larsen, Erik Lerkevang Grove, Manan Pareek, Steen Dalby Kristensen, Anne-Mette Hvas

**Affiliations:** 1 Department of Cardiology, Aarhus University Hospital, DK-8200, Aarhus N, Denmark; 2 Faculty of Health Sciences, Aarhus University, DK-8200, Aarhus N, Denmark; 3 Department of Clinical Biochemistry, Aarhus University Hospital, DK-8200, Aarhus N, Denmark; University of Leuven, BELGIUM

## Abstract

**Background:**

Recent studies suggest that the inflammation-associated protein calprotectin may be implicated in the pathogenesis of coronary artery disease (CAD). However, the impact of calprotectin levels on platelet aggregation in CAD patients has never been investigated.

**Objectives:**

We investigated the association between calprotectin levels and platelet aggregation in stable, high-risk CAD patients receiving aspirin as mono antiplatelet therapy. Furthermore, we aimed to investigate independent clinical and laboratory determinants of calprotectin levels.

**Methods:**

We performed a cross-sectional study including 581 stable, high-risk CAD patients. All patients received 75 mg aspirin daily as mono antiplatelet therapy. Platelet aggregation was assessed by 1) impedance aggregometry (Multiplate Analyzer) using arachidonic acid (AA) and collagen as agonists and by 2) the VerifyNow Aspirin Assay. Low-grade inflammation was evaluated by calprotectin, high-sensitive C-reactive-protein (hs-CRP) and interleukin-6. Platelet activation was assessed by soluble P-selectin, and cyclooxygenase-1 inhibition was evaluated by serum thromboxane B_2_, both measured by ELISA.

**Results:**

Calprotectin levels correlated positively with platelet aggregation according to Multiplate Analyzer (r=0.12, p=0.01). Additionally, calprotectin was positively associated with leukocytes (r=0.33, p<0.0001), hs-CRP (r=0.31, p<0.0001), interleukin-6 (r=0.28, p<0.0001), soluble P-selectin (r=0.10, p=0.02) and serum thromboxane B_2_ (r=0.10, p=0.02). Type 2 diabetes mellitus was an independent predictor of increased calprotectin levels (p=0.004), and trends were seen for body mass index (p=0.06) and smoking (p=0.07). Compliance with aspirin was confirmed by low serum thromboxane B_2_ levels in all patients (median [25%;75%]: 1.07 [0.52;1.87] ng/mL).

**Conclusion:**

Calprotectin levels correlated positively, though weakly, with platelet aggregation and activation as well as serum thromboxane B_2_ in high-risk, stable CAD patients treated with aspirin.

## Introduction

Inflammation plays an important role in the pathogenesis of atherosclerosis [1]. Coronary atherosclerosis is the underlying substrate of most coronary events, and rupture of an atherosclerotic plaque with exposure of the thrombogenic lipid-core promotes platelet adhesion followed by platelet activation and aggregation [2]. Platelet inhibition with aspirin continues to be the antiplatelet backbone in prevention and treatment of coronary artery disease (CAD) [3]. However, wide variability in the antiplatelet effect of aspirin has been reported, most likely reflecting the influence of genetic, biological and clinical factors [4,5].

Calprotectin, also known as myeloid-related protein 8/14, S100A8/A9 or calgranulin A/B, is an inflammation-associated protein [6], which is predominantly expressed by, and released from, myeloid cells on cellular activation [7,8]. Calprotectin plasma levels have primarily been investigated as a marker of inflammatory conditions such as inflammatory bowel disease and rheumatoid arthritis [9,10], but recent studies suggest that calprotectin may also be implicated in the pathogenesis of CAD [11–15]. Additionally, calprotectin has been identified as an early and sensitive biomarker with potential ability to discriminate between patients with acute coronary syndrome and patients with chronic, stable CAD [11,12,15]. Finally, elevated levels of calprotectin have been associated with increased risk of first and recurrent cardiovascular events [12,15,16].

Recent studies have suggested that increased levels of inflammatory markers, such as high-sensitive C-reactive protein (hs-CRP) and interleukin-6 (IL-6), may modify platelet aggregation and reduce the efficacy of antiplatelet drugs [17–21]. However, the impact of calprotectin levels on platelet aggregation in stable CAD patients has not been investigated. We hypothesized that high levels of calprotectin were associated with reduced effect of aspirin as indicated by increased platelet aggregation levels in stable, high-risk CAD patients. The aim of this study was to investigate the association between calprotectin levels and platelet aggregation in stable, high-risk CAD patients receiving aspirin as mono antiplatelet therapy. Furthermore, we aimed to investigate independent clinical and laboratory determinants of calprotectin levels.

## Methods

### Study population

We performed a cross-sectional study including 581 stable patients with angiographically documented CAD. Although stable, the study cohort represented a high-risk CAD population since all patients had either prior myocardial infarction, type 2 diabetes mellitus or both. All patients were recruited from the Western Denmark Heart Registry [22] and enrolled from February 2009 to January 2011. Fulfilment of the inclusion and exclusion criteria were checked in all patients’ medical records. The inclusion criteria were: a) age ≥ 18 years, b) significant CAD verified by prior percutaneous coronary intervention, coronary artery bypass grafting, or by a coronary angiography showing at least one 50% coronary luminal stenosis, c) patients with prior myocardial infarction: at least 12 months ago, myocardial infarction verified by electrocardiographic ST-segment elevation and/or elevated plasma troponin T (> 0.10 μg/L) together with plasma creatine kinase-MB (> 12 U/L). All diabetic patients were diagnosed with type 2 diabetes and treated with oral antidiabetic drugs and/or insulin. All non-diabetic patients had fasting plasma glucose levels < 7.0 mmol/L at the time of inclusion. The exclusion criteria were: a) ongoing treatment known to affect platelet function or coagulation (e.g. non-steroidal anti-inflammatory drugs, any antiplatelet drug or anticoagulants), b) any ischaemic vascular event, percutaneous coronary intervention, or coronary artery bypass grafting within the previous 12 months, c) platelet count < 120 x 10^9^/L or > 450 x 10^9^/L.

All patients included in the study were treated with 75 mg non-enteric coated aspirin once daily as mono antiplatelet therapy prior to and during the study.

The study was conducted in agreement with the Helsinki-II-declaration. It was approved by The Central Denmark Region Committees on Health Research (M-2009-0110) and by the Danish Data Protection Agency. All patients gave written informed consent.

### Compliance

To optimize compliance and uniform pharmacokinetics, all patients received a pill box with seven tablets of 75 mg non-enteric coated aspirin (Hjerdyl, Sandoz, Denmark). This was to ensure that all patients included in the study received the exact same aspirin dose and preparation prior to and at the time of blood sampling. On the day of blood sampling, patients were instructed to ingest aspirin exactly one hour before blood sampling. Compliance with aspirin was optimized by confirmation of empty pill boxes along with aspirin intake questioning, and finally, measurement of serum thromboxane B_2_ (S-TXB_2_) was performed for all patients.

### Laboratory investigations

#### Blood sampling

Blood samples were obtained from the antecubital vein with patients in supine position after 30 minutes of rest using vacuum tubes, a large bore needle (19 G), and a minimum of stasis.

#### Haematology

Blood samples for haematological analyses, including haemoglobin, leukocyte count and platelet count, were collected in 3.0 mL tubes containing EDTA (Terumo, Leuven, Belgium) and analysed within 90 minutes of sampling. Haematological parameters were measured with the Sysmex XE-2100 haematology analyser (Sysmex, Kobe, Japan).

#### Platelet aggregation tests

Platelet aggregation was evaluated with two different instruments using whole blood: impedance aggregometry (Multiplate Analyzer, Roche, Hvidovre, Denmark) and the VerifyNow Aspirin Assay (Accumetrics Inc., San Diego, CA, USA).

Impedance aggregometry is based on platelet adhesion and aggregation on two electrodes, resulting in an increase of electrical resistance, which is converted into arbitrary aggregation units (AU). The area under the aggregation curve (AUC) is used to express the aggregation response over the measured time (AU*min). For impedance aggregometry analyses, 3.0 mL tubes containing hirudin 25 μg/mL (Terumo, Lueven, Belgium) were utilised. Platelet aggregation was induced using arachidonic acid (AA) 1.0 mM (ASPI test, Triolab AS, Brøndby, Denmark) or collagen 1.0 μg/mL (Horm, Medinor, Nycomed, Austria). Blood samples rested for at least 30 minutes before analysis but no longer than 120 minutes. In case of a deviation > 20% between the two impedance curves, the sample was re-analysed.

VerifyNow is based on turbidimetric optical detection. Whole blood is directed to mixing chambers containing fibrinogen-coated beads and AA 1.0 mM as agonist and platelets, which are not adequately inhibited by aspirin become activated by AA and agglutinate out of the solution. Light transmittance through the sample is measured and converted into arbitrary aspirin reaction units (ARU). For VerifyNow analyses, blood was collected in 2.7 mL tubes containing 3.2% sodium citrate (Terumo, Lueven, Belgium). Blood samples rested for at least 30 minutes before analysis but no longer than 120 minutes. The reproducibility of the platelet aggregation tests has previously been reported by our group [23].

### Calprotectin and other inflammatory markers

Blood for calprotectin analysis was collected in non-siliconized 5.0 mL tubes (Terumo, Leuven, Belgium) without anticoagulants. The blood was allowed to clot for one hour at 37°C before serum was separated by centrifugation at 2600 *g* for 10 minutes. Serum was stored at −80°C until analysis. Serum calprotectin was measured using enzyme-linked immunosorbent assay (ELISA) (MRP 8/14 Calprotectin, Bühlmann, Schönenbuch, Switzerland). The first four steps of the ELISA protocol were performed using a JANUS Automated workstation (PerkinElmer, Waltham, MA, USA). Duplicate measurements were performed on 40 samples (coefficient of variation = 2.5%), and single measurements were performed in the remaining 541 samples.

Blood for hs-CRP analysis (KoneLab 30i, ILS Laboratories Scandinavia, Allerød, Denmark) was collected in 3.0 mL lithium-heparin tubes containing separating gel (Terumo, Leuven, Belgium). The measurement interval for hs-CRP was 0.2–10.0 mg/L. A total of 493 (85%) of the 581 patients were included in the hs-CRP analyses; 28 patients had hs-CRP levels >10.0 mg/L, and hs-CRP was not measured in 57 patients due to a change in laboratory procedures regarding measurements of hs-CRP during the study. Hs-CRP values were missing for three patients.

Blood for IL-6 analysis (cobas 6000 analyser, E module, Roche, Mannheim, Germany) was collected in non-siliconized 5.0 mL tubes (Terumo, Leuven, Belgium) without anticoagulants. The blood was allowed to clot for one hour at 37°C before serum was separated by centrifugation at 2600 *g* for 10 minutes. Serum was stored at −80°C until analysis.

#### Soluble P-selectin and serum thromboxane B2

Concentrations of soluble P-selectin (sP-selectin) and serum thromboxane B_2_ were measured using ELISA (R&D Systems, Minneapolis, USA and Thromboxane B_2_ EIA Kit, Cayman Chemical, Michigan, USA) as previously described [24].

### Ethics statement

The study was conducted in agreement with the Helsinki-II-declaration. It was approved by The Central Denmark Region Committees on Health Research (M-2009-0110) and by the Danish Data Protection Agency. All patients gave written informed consent.

### Statistics

If normally distributed, continuous data are presented as mean and standard deviation (SD), if not as median and interquartile range or log-transformed to obtain normal distribution. Differences between two unpaired groups were tested with a two-sided t-test if data were normally distributed and, if not, the Mann-Whitney test was used. Proportions between two groups were compared using Fischer’s exact test and presented as absolute counts and percentages. Correlations were calculated using Spearman’s rank coefficient. Multiple linear regression analyses were used to identify independent determinants of calprotectin and platelet aggregation. A two-sided p-value < 0.05 was considered statistically significant. Data were registered in Epidata version 3.1 (EpiData Association, Denmark). Statistical analyses were performed using Stata version 11.0 (StataCorp, College Station, TX, USA), and graphs were made using GraphPad Prism version 5.0 (GraphPad Software, San Diego, CA, USA).

## Results

### Study population

Clinical and biochemical characteristics of the study population are shown in Tables 1 and 2. The study population consisted of stable CAD patients with a relatively high-risk profile, since 533 (92%) of the patients had a history of myocardial infarction, 145 (25%) had type 2 diabetes, and 100 (17%) had both. Compliance with aspirin was confirmed by low serum thromboxane B_2_ levels in all patients (median [25%;75%]: 1.07 [0.52;1.87], range 0.02–26.44 ng/mL).

**Table 1:** Baseline characteristics of the study population, n = 581.

Age, years	64±9
Body mass index, kg/m^2^	28 ±4
Males	460 (79)
Current smokers	122 (21)
Blood pressure, systolic, mm Hg	142±20
Blood pressure, diastolic, mm Hg	82±11
*Morbidity*	
Prior percutaneus coronary intervention	562 (97)
Prior myocardial infarction	533 (92)
Prior coronary artery bypass grafting	49 (8)
Prior stroke	25(4)
Type 2 diabetes mellitus	145 (25)
*Medication*	
Aspirin	581 (100)
Statins	533 (92)
Beta-blockers	440 (76)
ACE inhibitors	265 (46)
Angiotensin receptor blockers	80 (14)
Calcium antagonists	111 (19)
Diuretics	147 (25)
Proton pump inhibitors	65 (11)
Insulin*	47 (32)
Oral antidiabetic medication*	127 (88)

Data is presented as mean±SD or n (%). B: blood, S: serum, P: plasma, ACE: Angiotensin converting enzyme.

*out of 145 patients with type 2 diabetes.

**Table 2:** Baseline bichemical characteristics of the study population, n = 581.

		*reference interval*
B-Leukocyte count	7.0±1.9	3.5–10.0 10^9^/L
B-Haemoglobin	8.9±0.7	7.3–10.5 mmol/L*
B-Platelet count	231±59	145–400 10^9^/L*
S-Calprotectin	1.1 (0.8;1.6)	< 3.5 μg/mL**
P-High-sensitive C-reactive protein	0.8 (0.4;1.6)	0.2–4.1 μg/mL
S-Interleukin-6	2.2 (1.5;3.4)	< 7 pg/mL**

Data is presented as mean ±SD or median (25%;75%). B: blood, S: serum, P: plasma.

*Reference interval for both men and women.

**In healthy individuals. No established reference intervals for stable CAD patients.

In order to evaluate baseline characteristics and biochemical values according to platelet aggregation levels, patients were divided into two subgroups on the basis of the median (163 AU*min) of AA-induced platelet aggregation using the Multiplate Analyzer (Table 3). Thus, in Table 3, patients with high platelet aggregation had aggregation levels ≥ the median of 163 AU*min and patients with low platelet aggregation had aggregation levels < the median of 163 AU*min. Patients with high platelet aggregation levels had significantly higher calprotectin levels than patients with low aggregation. Also leukocyte counts, platelet counts, hs-CRP and IL-6 levels were elevated in the high platelets aggregation group. Furthermore, high platelet aggregation levels were significantly associated with type 2 diabetes, smoking and treatment with calcium antagonists and diuretics.

**Table 3:** Baseline characteristics of the study population according to platelet aggregation using Multiplate Analyzer, 1.0 mM arachidonic acid, n = 581.

	<163 AU*min	≥163 AU*min	*p-value*
	*n = 283*	*n = 298*	
Age, years	64±9	64±10	0.25
Body mass index, kg/m^2^	27±4	28±5	0.06
Males	227 (80)	227 (78)	0.48
Current smokers	45 (16)	75 (26)	0.01
Blood pressure, systolic, mm Hg	142±20	141±20	0.65
Blood pressure, diastolic, mm Hg	83±12	82±10	0.09
*Morbidity*			
Prior percutaneus coronary intervention	275 (97)	281 (96)	0.64
Prior myocardial infarction	265 (94)	265 (91)	0.22
Prior coronary artery bypass grafting	23 (8)	26 (9)	0.77
Prior stroke	13 (5)	12 (4)	0.84
Type 2 diabetes mellitus	57 (20)	88 (30)	0.01
*Medication*			
Aspirin	282 (100)	292 (100)	1.00
Statins	256 (91)	272 (93)	0.36
Beta-blockers	216 (77)	219 (75)	0.70
ACE inhibitors	128 (45)	136 (47)	0.80
Angiotensin receptor blockers	43 (15)	36 (12)	0.33
Calcium antagonists	40 (14)	68 (23)	0.01
Diuretics	53 (19)	90 (31)	0.001
Proton pump inhibitors	29 (10)	35 (12)	0.60
Insulin*	16 (28)	30 (34)	0.47
Oral antidiabetic medication*	48 (84)	78 (89)	0.46

Data is presented as mean±SD, n(%) or median 25%;75%). B: blood, S: serum, P: plasma, ACE: Angiotensin converting enzyme. Patients were divided into two subgroups on the basis of the median (163 AU*min) of AA-induced platelet aggregation using the Multiplate Analyzer.

*Out of 145 patients with type 2 diabetes.

### Calprotectin and platelet aggregation

**Fig 1:**
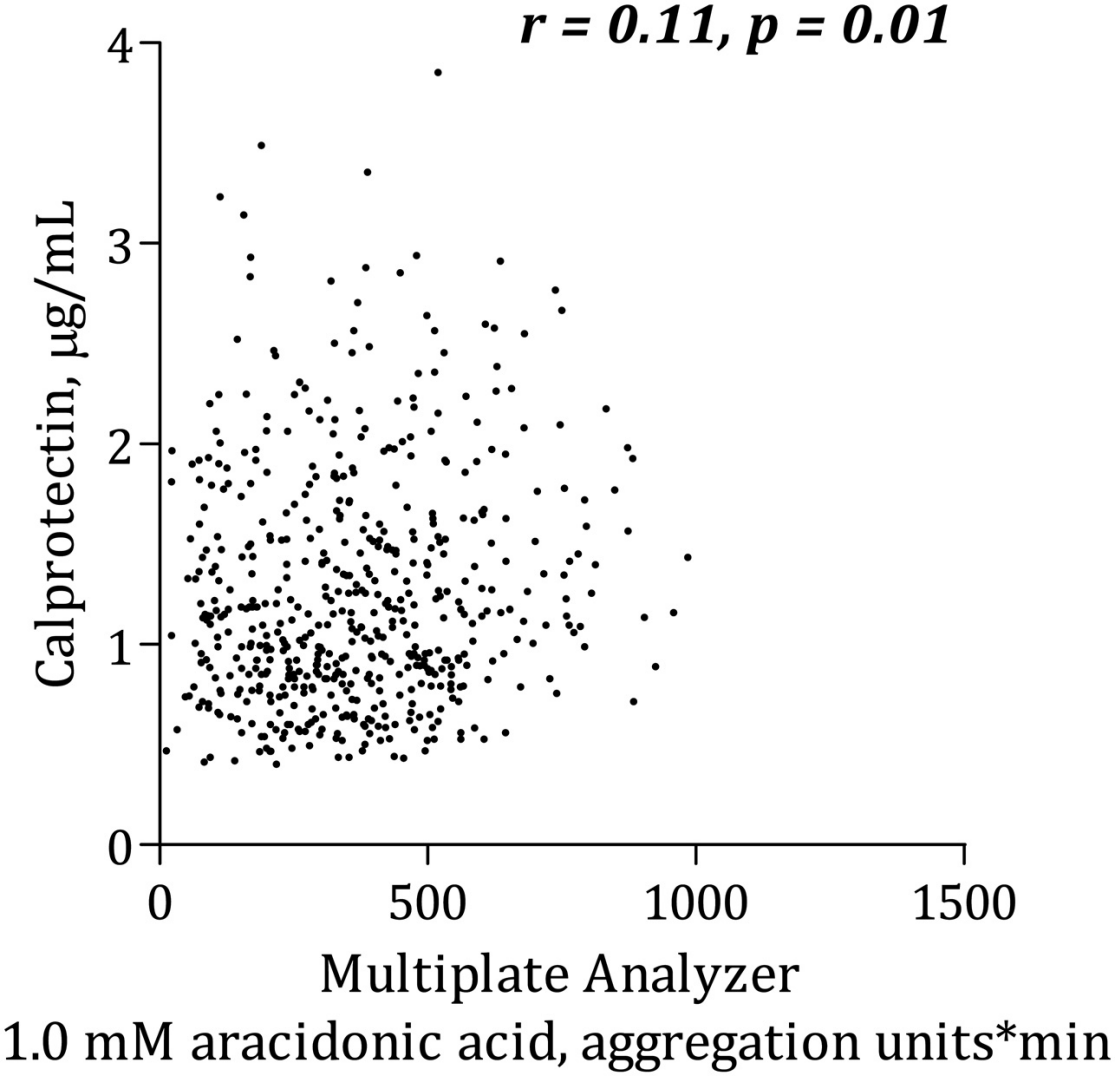
Correlation between calprotectin and platelet aggregation induced by arachidonic acid using Multiplate Analyzer.

**Fig 2:**
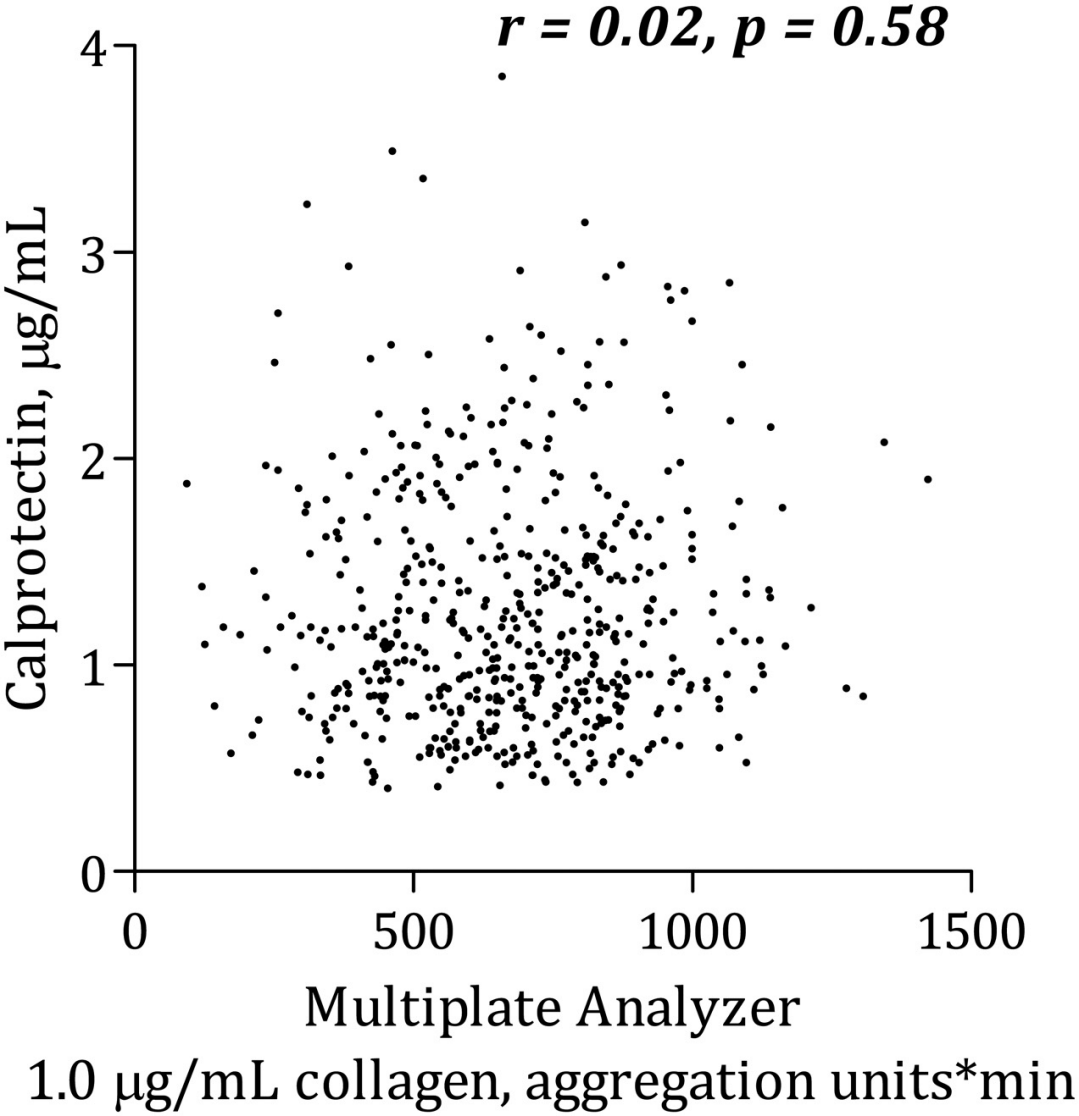
Correlation between calprotectin and platelet aggregation induced by collagen using Multiplate Analyzer.

**Fig 3:**
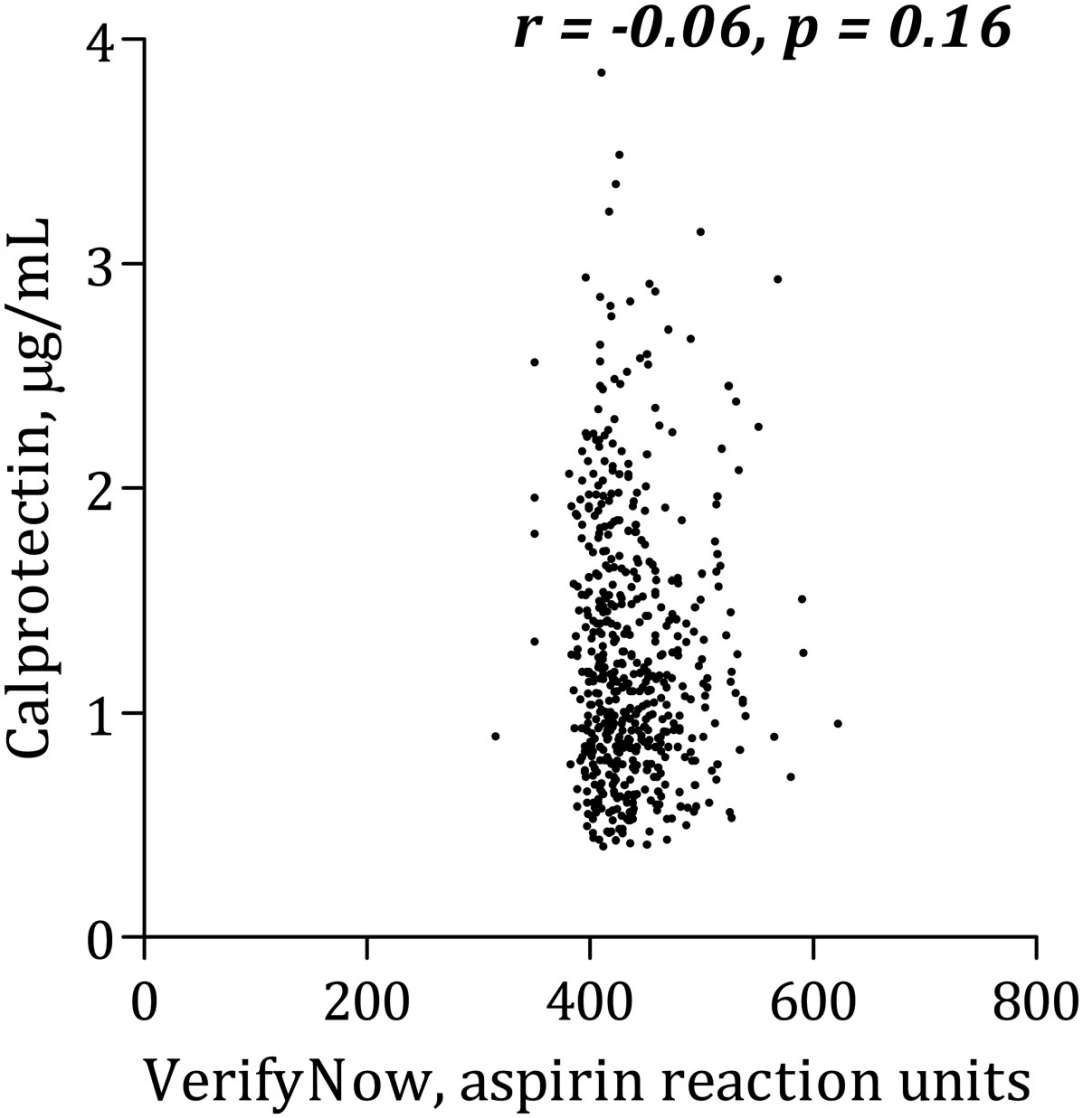
Correlation between calprotectin and platelet aggregation induced by arachidonic acid using VerifyNow.

**Fig 4:**
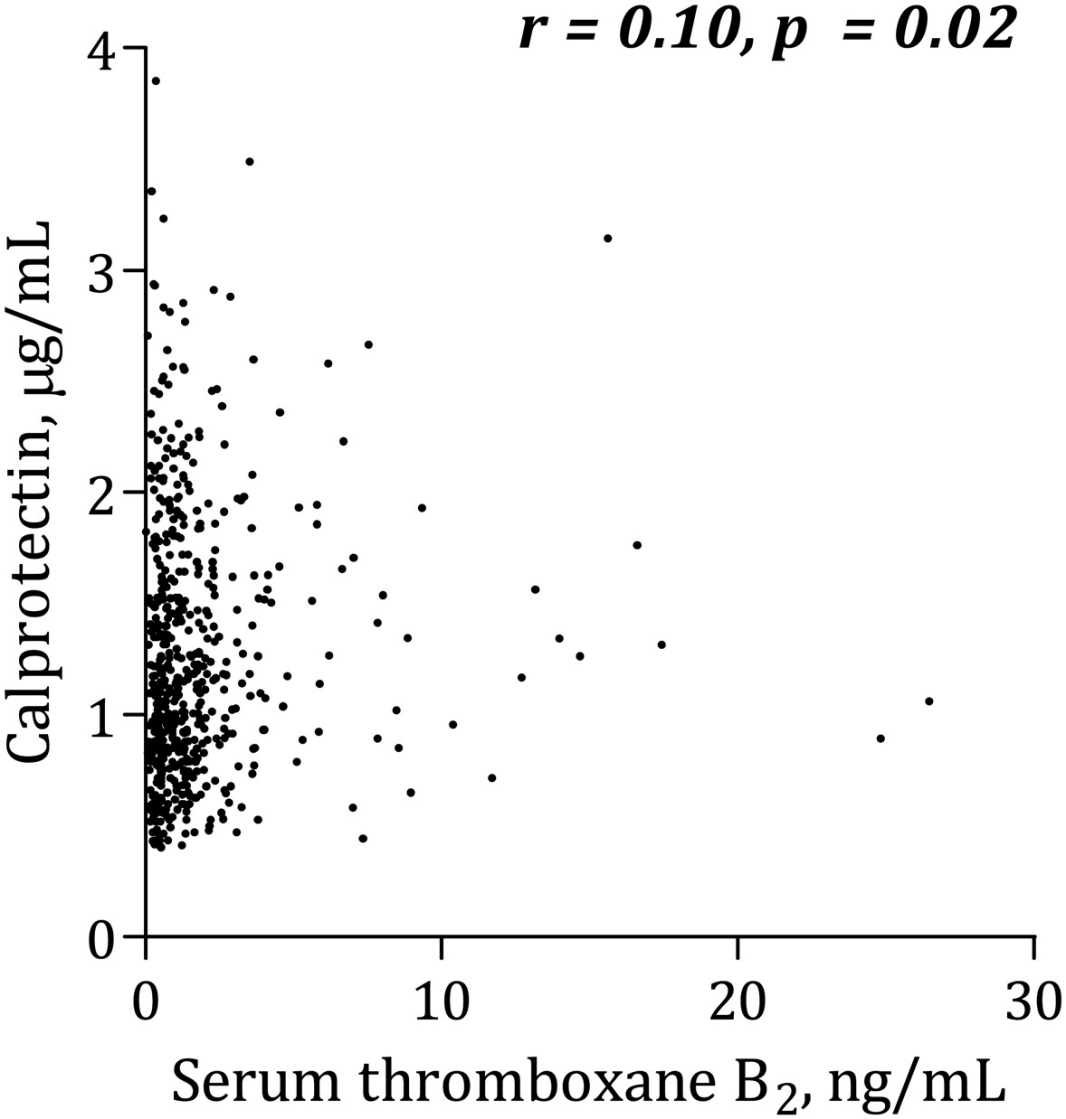
Correlation between calprotectin and serum thromboxane B_2_.

**Fig 5:**
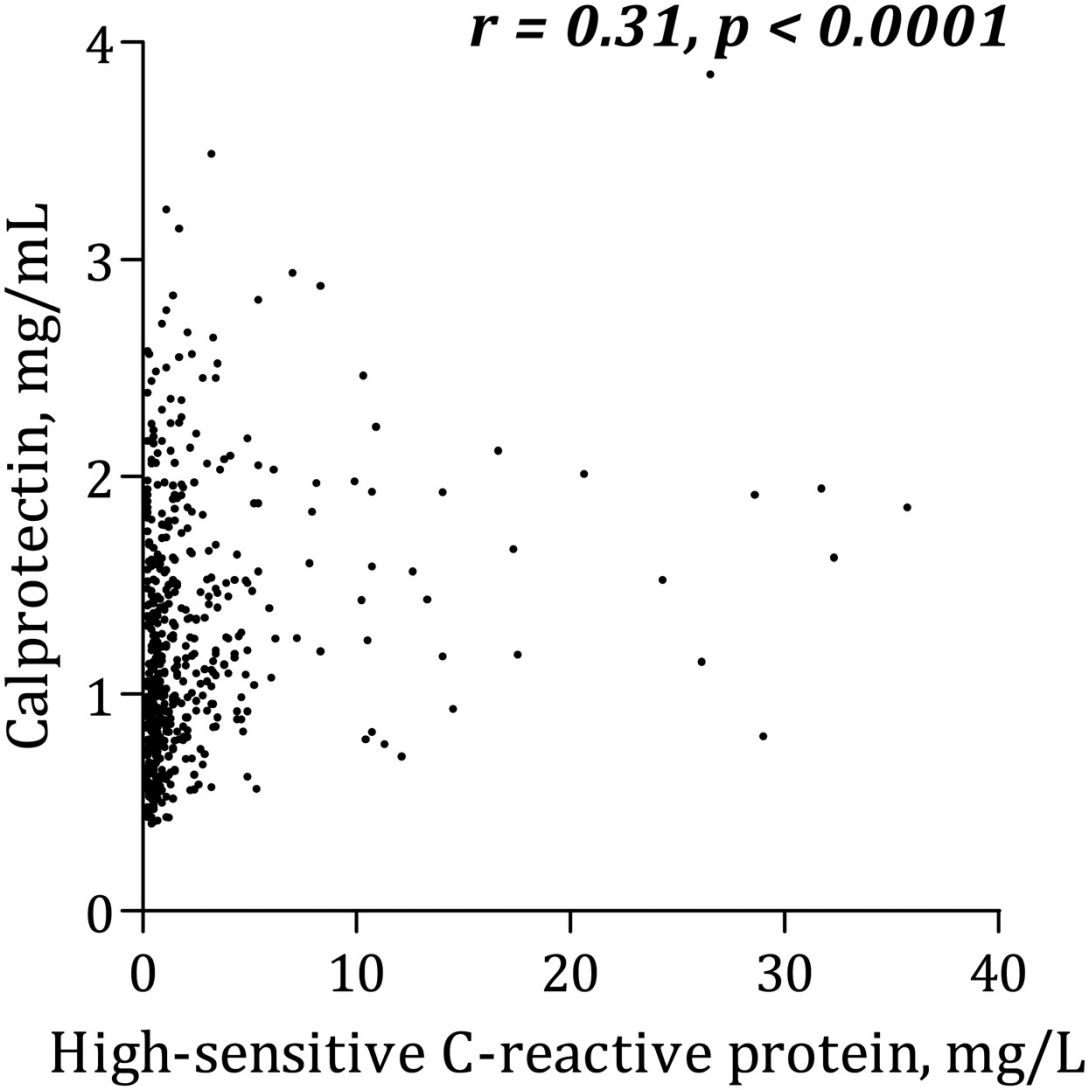
Correlation between calprotectin and C-reactive protein.

**Fig 6:**
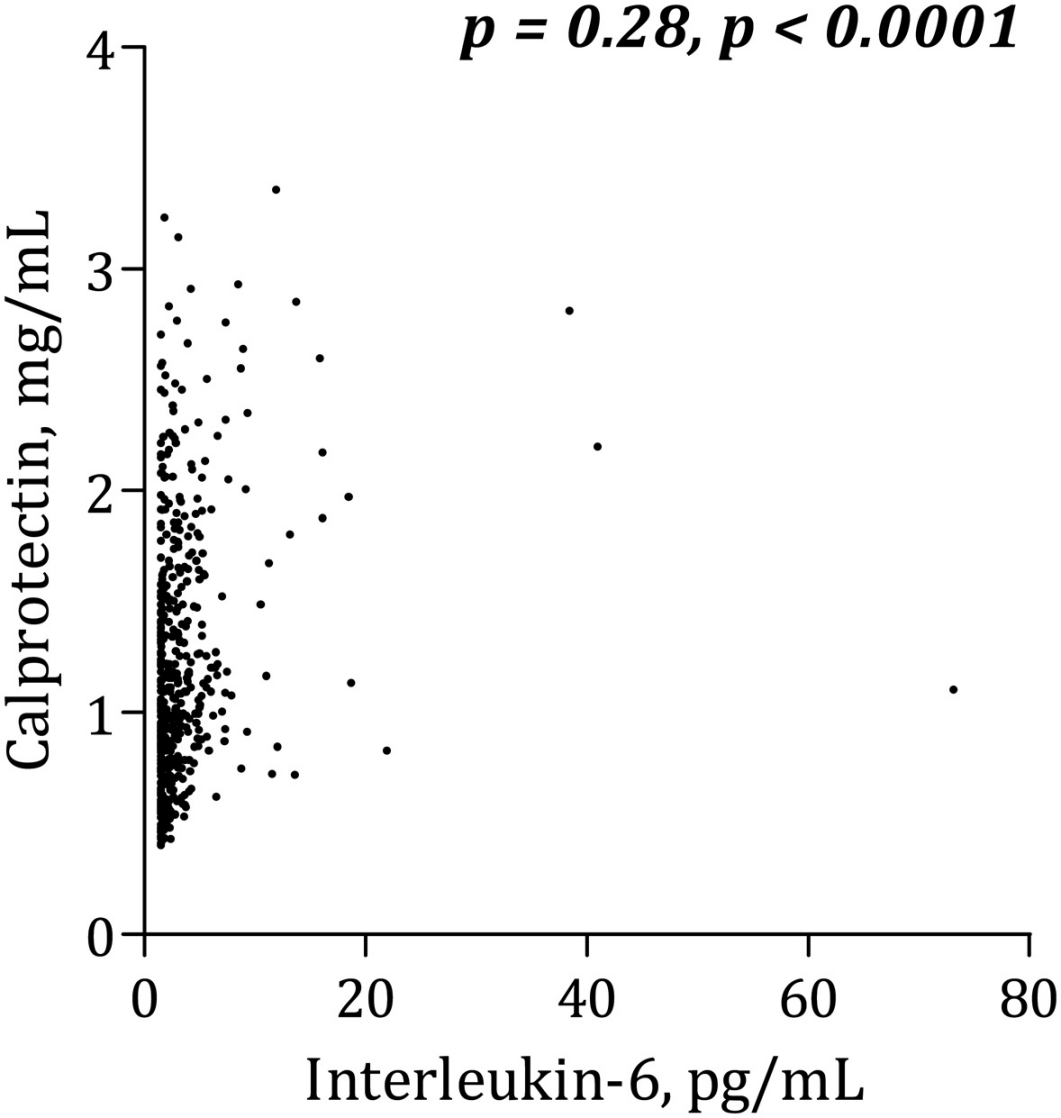
Correlation between calprotectin and interleukin-6.

Calprotectin was positively, though weakly, correlated with AA-induced platelet aggregation according to Multiplate Analyzer (Fig 1). There was no significant association between calprotectin and collagen-induced Multiplate Analyzer platelet aggregation (Fig 2) or VerifyNow (Fig 3). Calprotectin was positively associated with serum thromboxane B_2_ (Fig 4), sP-selectin (r = 0.10, p = 0.02), leukocytes (r = 0.33, p < 0.0001), hs-CRP (Fig 5) and IL-6 (Fig 6).

After adjusting for traditional risk factors (gender, age, type 2 diabetes, smoking status and body mass index) in a linear multivariate regression analysis, calprotectin no longer predicted increased AA-induced platelet aggregation according to the Multiplate Analyzer (p = 0.10). Similar results were found with collagen-induced platelet aggregation and VerifyNow.

### Independent determinants of calprotectin

In order to identify clinical and demographical determinants of calprotectin, we performed linear multivariate regression analysis including age, gender, smoking, body mass index, prior myocardial infarction and type 2 diabetes as independent variables (Table 4). Type 2 diabetes was an independent determinant of calprotectin (p = 0.004), and trends were seen for body mass index (p = 0.06) and smoking (p = 0.07). Calprotectin levels were increased by 17% in patients with type 2 diabetes compared with non-diabetes patients, and by 9% in smokers compared with non-smokers, however, the latter did not reach statistical significance (Table 4).

**Table 4:** Calprotectin and clinical and demographical predictors, n = 581.

	*beta*	*95% CI*	*p-value*
Age, years	1.00	0.95;1.14	0.72
Female gender	1.04	0.95;1.14	0.42
Current smoking	1.09	0.99;1.20	0.07
Body mass index, kg/m^2^	1.01	1.00;1.02	0.06
Prior myocardial infarction	0.95	0.81;1.12	0.56
Type 2 diabetes mellitus	1.17	1.05;1.29	0.004

Multiple linear regression analysis. CI: confidence interval.

## Discussion

This is the first study to investigate the association between calprotectin and platelet aggregation in stable, high-risk CAD patients. The main findings of the study were that 1) increased levels of calprotectin were associated with AA-induced platelet aggregation according to Multiplate Analyzer and serum thromboxane B_2_, and 2) type 2 diabetes was an independent determinant of increased calprotectin levels.

### Calprotectin and platelet aggregation

We found positive associations between calprotectin levels and AA-induced platelet aggregation according to Multiplate Analyzer. Furthermore, positive correlations between levels of calprotectin and serum thromboxane B_2_ and sP-selectin were found. This may suggest that calprotectin could influence on both platelet activation and subsequent aggregation; however, all correlations were rather weak. In a previous study including part of the present study population, we demonstrated that high levels of hs-CRP were associated with increased platelet aggregation in stable CAD patients [19]. In the present study, we have demonstrated that calprotectin was significantly associated with both hs-CRP and IL-6.

Inflammation is an important player in thrombosis [25,26]. Calprotectin has been shown to induce a thrombogenic, inflammatory response in endothelial cells by increasing the transcription of pro-inflammatory chemokines and adhesion molecules [27], which may secondarily activate platelets, possibly through expression of P-selectin on endothelial cells. However, a direct effect of calprotectin on platelet aggregation has not been established. Recently, in an experimental study, Wang et al. reported that the subunit MRP14 or S100A9 is released by platelets and that the subunit is involved in the molecular pathways leading to thrombus formation through CD36 [28]. Other inflammatory markers have also been proposed to influence platelets; CRP may indirectly affect platelet reactivity through preceding FcƴRIIa-dependent monocyte-activation [25] and CRP-induction of macrophage-derived tissue factor generation [26]. These pathways may partly explain the association between hs-CRP and reduced antiplatelet effect of aspirin [19], and the association between hs-CRP and calprotectin, the latter being demonstrated in the present study.

### Calprotectin and cardiovascular risk factors

Diabetes, obesity and smoking are traditional cardiovascular risk factors, which have previously been shown to associate with elevated levels of calprotectin [29]. In the present study, type 2 diabetes was an independent determinant of increased calprotectin levels, and trends were seen for body mass index and smoking. Thus, our results extend previous findings in healthy individuals showing that calprotectin levels are positively influenced by smoking and body mass index [16].

Diabetes is considered a pro-thrombotic state, probably explained by disturbances in various steps of thrombus formation, which may lead to increased platelet activation and aggregation [30]. Several mechanisms, including chronic low-grade inflammation, have been suggested as potential mechanisms leading to platelet hyper reactivity, hyper coagulability and compromised fibrinolysis [31,32]. In our study, calprotectin levels were increased by 17% in CAD patients with type 2 diabetes compared with non-diabetes patients. Our results extend the study by Peng et al. showing elevated calprotectin levels in patients with type 2 diabetes and CAD compared with type 2 diabetes patients without CAD [33]. Additionally, the authors found that calprotectin levels correlated positively with the severity of CAD and with carotid intima-media thickness in diabetes patients without clinically overt CAD [33]. Catalan et al. reported increased levels of calprotectin in patients with obesity associated type 2 diabetes and found that calprotectin levels decreased following weight loss [34].

In our study, type 2 diabetes was an independent predictor of increased calprotectin levels, whereas the association between calprotectin and increased platelet aggregation was less pronounced. The association between diabetes and chronic low-grade inflammation may explain the increased levels of calprotectin in diabetic patients. To the best of our knowledge, no studies have demonstrated a relationship between increased calprotectin levels and a reduced antiplatelet effect of aspirin. Most likely, calprotectin only has a modest influence on platelet aggregation.

Body mass index correlated significantly with increased calprotectin levels, thus extending previous findings in healthy individuals [16,35]. However, when adjusted for gender, age, prior myocardial infarction, diabetes and smoking, only a trend was seen. This may be partially explained by the influence of type 2 diabetes; calprotectin levels have been reported to be higher in obese than in non-obese non-diabetic subjects [35–37], whereas calprotectin levels did not differ between obese and non-obese patients with diabetes [35,36], which is in line with our results (data not shown). This may suggest that the mechanisms involved in augmented calprotectin production in obesity and type 2 diabetes overlap.

Calprotectin is primarily released from neutrophils and monocytes/macrophages [29]. Smoking is known to stimulate granulopoiesis and calprotectin production, and previous studies have demonstrated a strong relationship between blood neutrophil counts and calprotectin concentrations [16,36]. In the present study, smoking was associated with elevated levels of calprotectin, however the association became non-significant when adjusted for gender, age, prior myocardial infarction, diabetes and body mass index.

Increased calprotectin concentrations have been associated with atherosclerosis [13,38] and plaque vulnerability [13,39], severity of CAD [33], acute myocardial infarction [11,14,40], and increased risk of future cardiovascular events in both healthy individuals [12,16] and patients with cardiovascular disease [15,40]. Calprotectin has been associated with endotoxin-induced cardiomyocyte dysfunction [41] as well as amplification of inflammation [42]. This interplay may be involved in the development of CAD and acute coronary syndromes [43].

In our study, calprotectin levels were higher in patients without prior myocardial infarction than in patients with prior myocardial infarction. However, the association became non-significant after adjustment for gender, age, diabetes, smoking and body mass index. Thus, our results suggest that in stable CAD patients, prior myocardial infarction is not a major determinant of calprotectin levels.

### Strengths and limitations

The overall strength of this study is the inclusion of a large study population with stable CAD. Although we studied high-risk CAD patients, our study population is similar to a large fraction of everyday CAD patients, and the study results thus have high external validity. Pre-analytical procedures, aspirin dosing and the interval from aspirin intake to platelet aggregation analysis were strictly standardized. Furthermore, compliance with aspirin was confirmed in all patients by low serum thromboxane B_2_ levels.

In this observational study, we were not able to establish causality between calprotectin and platelet aggregation levels. Further limitations include that we did not measure neutrophil count or the subunit MRP 8.

### Conclusion

Although weak, the present study demonstrated for the first time positive correlations between calprotectin levels, platelet aggregation and serum thromboxane B_2_ in stable, high-risk CAD patients treated with low-dose aspirin.
